# *Aegilops tauschii* genome assembly Aet v5.0 features greater sequence contiguity and improved annotation

**DOI:** 10.1093/g3journal/jkab325

**Published:** 2021-09-13

**Authors:** Le Wang, Tingting Zhu, Juan C Rodriguez, Karin R Deal, Jorge Dubcovsky, Patrick E McGuire, Thomas Lux, Manuel Spannagl, Klaus F X Mayer, Patricia Baldrich, Blake C Meyers, Naxin Huo, Yong Q Gu, Hongye Zhou, Katrien M Devos, Jeffrey L Bennetzen, Turgay Unver, Hikmet Budak, Patrick J Gulick, Gabor Galiba, Balázs Kalapos, David R Nelson, Pingchuan Li, Frank M You, Ming-Cheng Luo, Jan Dvorak

**Affiliations:** 1 Department of Plant Sciences, University of California, Davis, Davis, CA 95616, USA; 2 Plant Genome and Systems Biology, Helmholtz Zentrum München, Munich 85764, Germany; 3 Donald Danforth Plant Science Center, St. Louis, MO 63132, USA; 4 Division of Plant Sciences, University of Missouri, Columbia, Columbia, MO 65211, USA; 5 Crop Improvement and Genetics Research Unit, United States Department of Agriculture-Agricultural Research Service, Western Regional Research Center, Albany, CA 94710, USA; 6 Institute of Bioinformatics, University of Georgia, Athens, GA 30602, USA; 7 Institute of Plant Breeding, Genetics and Genomics, University of Georgia, Athens, GA 30602, USA; 8 Department of Crop and Soil Sciences, University of Georgia, Athens, GA 30602, USA; 9 Department of Plant Biology, University of Georgia, Athens, GA 30602, USA; 10 Department of Genetics, University of Georgia, Athens, GA 30602, USA; 11 Ficus Biotechnology, Ostim Teknopark, Ankara 06374, Turkey; 12 Department of Genomics and Genome Editing, Montana BioAg Inc., Missoula, MT 59801, USA; 13 Department of Biology, Concordia University, Montreal, Quebec H3G 1M8, Canada; 14 Department of Biological Resources, Centre for Agricultural Research, Eötvös Loránd Research Network, H-2462 Martonvásár, Hungary; 15 Department of Environmental Sustainability, IES, Hungarian University of Agriculture and Life Sciences, H-8360 Keszthely, Hungary; 16 Department of Microbiology, Immunology and Biochemistry, University of Tennessee Health Science Center, Memphis, TN 38163, USA; 17 Ottawa Research and Development Centre, Agriculture and Agri-Food Canada, Ottawa, Ontario K1A 0C5, Canada

**Keywords:** disease resistance, miRNA, phasiRNA, hc-siRNA, tRNA, tRF, transposable elements, optical map, Pacific Biosciences

## Abstract

*Aegilops tauschii* is the donor of the D subgenome of hexaploid wheat and an important genetic resource. The reference-quality genome sequence Aet v4.0 for *Ae. tauschii* acc. AL8/78 was therefore an important milestone for wheat biology and breeding. Further advances in sequencing acc. AL8/78 and release of the Aet v5.0 sequence assembly are reported here. Two new optical maps were constructed and used in the revision of pseudomolecules. Gaps were closed with Pacific Biosciences long-read contigs, decreasing the gap number by 38,899. Transposable elements and protein-coding genes were reannotated. The number of annotated high-confidence genes was reduced from 39,635 in Aet v4.0 to 32,885 in Aet v5.0. A total of 2245 biologically important genes, including those affecting plant phenology, grain quality, and tolerance of abiotic stresses in wheat, was manually annotated and disease-resistance genes were annotated by a dedicated pipeline. Disease-resistance genes encoding nucleotide-binding site domains, receptor-like protein kinases, and receptor-like proteins were preferentially located in distal chromosome regions, whereas those encoding transmembrane coiled-coil proteins were dispersed more evenly along the chromosomes. Discovery, annotation, and expression analyses of microRNA (miRNA) precursors, mature miRNAs, and phasiRNAs are reported, including miRNA target genes. Other small RNAs, such as hc-siRNAs and tRFs, were characterized. These advances enhance the utility of the *Ae. tauschii* genome sequence for wheat genetics, biotechnology, and breeding.

## Introduction

*Aegilops tauschii* (2*n* = 2*x* = 14, genomes DD) is a widely distributed goatgrass with a natural range from East Turkey to China and West Pakistan (van Slageren 1994). The species consists of two major phylogenetic lineages, designated as 1 and 2, which approximate *Ae. tauschii* ssp. *tauschii and Ae. tauschii* ssp. *strangulata*, respectively (Wang *et al.* 2014a).

*Aegilops tauschii* is the donor of the D subgenome of hexaploid wheat (*Triticum aestivum*, subgenomes AABBDD) (Kihara 1944; McFadden and Sears 1946). The bulk of the wheat D subgenome was contributed by lineage 2 (Wang *et al.* 2014a). The wheat D subgenome harbors genes responsible for the suitability of wheat flour for making bread and related products (Payne 1987) and resistance to diseases (Gill *et al.* 1986). *Aegilops tauschii* contributed environmental plasticity and stress tolerance to hexaploid wheat (Dubcovsky and Dvorak 2007) and is therefore an important genetic resource for wheat. The development of genomic resources for *Ae. tauschii* is critical for the use of this wheat progenitor in wheat studies and improvement.

*Aegilops tauschii* has a large (>4.2 Gb) and repeat-rich (84.4%) genome (Luo *et al.* 2017; Zhao *et al.* 2017). Most of the *Ae. tauschii* repeated sequences amplified in the past 2 million years (Dai *et al.* 2018a) and are highly homogeneous (Luo *et al.* 2017), which made sequencing this genome challenging. Two reference-quality genome sequences have been reported for *Ae. tauschii* acc. AL8/78 (lineage 2, Armenia) (Luo *et al.* 2017; Zhao *et al.* 2017). The sequence reported by Zhao *et al.* (2017) was built on contractual basis by NRGene Inc. using their proprietary DeNovoMAGIC assembler. Luo *et al.* (2017) used a combination of techniques to produce the AL8/78 genome sequence. A physical map was first constructed from bacterial artificial chromosome (BAC) clones (Luo *et al.* 2013) and a minimum tiling path across 42,822 clones was sequenced with an Illumina short-read sequencing platform. That sequence was merged with (1) a sequence assembled from whole-genome shotgun (WGS) Illumina reads built by NRGene Inc. with the DeNovoMAGIC assembler for the project (Luo *et al.* 2017) and (2) mega-reads built from Pacific Biosciences (PacBio) reads (Zimin *et al.* 2017). Scaffolding and super-scaffolding were guided by three genome-wide optical maps, one for *Ae. tauschii* acc. AL8/78, one for acc. CIae 23 (lineage 2, west Caspian Iran), and one for the wheat D subgenome (Luo *et al.* 2017). Scaffolds and super-scaffolds were anchored on a genetic map built using the *Ae. tauschii* 10-K Illumina Infinium single-nucleotide-polymorphism (SNP) assay (Luo *et al.* 2013). The resulting assembly (Aet v4.0) consisted of 4025 Mb in seven pseudomolecules and 200 Mb in unassigned scaffolds.

The three optical maps used to guide and validate the Aet v4.0 assembly were constructed with a chemistry that utilizes a single-strand restriction endonuclease for nicking DNA (Lam *et al.* 2012; Hastie *et al.* 2013). The nicks were labeled, repaired, and DNA molecules were stained. Chance clustering of nick sites produces fragile regions in DNA molecules, which are then prone to breaking during sample manipulation. Optical contigs therefore tend to terminate in these regions. Optical contigs also tend to terminate in blocks of heterochromatin, which often consist of low-complexity, tandem repeats. Polymorphism for restriction sites and heterochromatin may alter the locations of gaps on an optical map. The three optical maps employed for assembly Aet v4.0 were for genomes from *Ae. tauschii* lineage 2. Phylogenetic comparisons predict that accessions from lineage 1 will differ from those of lineage 2 in the distribution of some restriction sites and heterochromatic blocks. The distribution of gaps on the optical maps for accessions of lineage 1 is therefore expected to differ from the distribution of gaps on optical maps for accessions of lineage 2. Hence, optical maps for two *Ae. tauschii* accessions from lineage 1 were constructed here and used for further validation of super-scaffold in Aet v4.0.

Some 83,130 protein-coding genes, 39,635 high-confidence (HC), and 43,495 low-confidence (LC), were annotated in Aet 4.0 (Luo *et al.* 2017), but small RNA (sRNA) encoding genes were not characterized. Some sRNAs are important for transcriptional and post-transcriptional regulation of gene expression. These are short regulatory RNAs, between 18 and 34 nucleotides, involved in a wide variety of biological processes, from development to stress responses. MicroRNAs (miRNAs) and tRNA-derived fragments (tRFs) are derived from single-strand RNA precursors with a specific secondary structure. MicroRNAs are typically 21 or 22 nt long, and their precursors are hairpin RNAs transcribed by RNA Polymerase II (Axtell 2013). The tRFs are 19 to 34 nt long and their tRNA precursors are clover shaped and RNA Polymerase III dependent (Martinez *et al.* 2017). The siRNAs are amplified by RNA-dependent RNA polymerase (RDR) enzymes, are derived from double-stranded RNA precursors, and can be divided into phased siRNAs (phasiRNAs) and heterochromatic siRNAs (hc-siRNAs). PhasiRNAs are 21 or 24 nt long, originate from coding or noncoding regions, and require a trigger microRNA for their biogenesis. PhasiRNAs are typically triggered by 22-nt miRNAs (Liu *et al.* 2020). Hc-siRNAs are derived from repetitive regions, are RNA Polymerase IV dependent, and are predominantly 24 nt long (Axtell 2013). Each class of mature sRNA is loaded onto an ARGONAUTE (AGO) family protein and directs transcriptional or post-transcriptional gene silencing in a sequence-specific manner.

Here, we report sequence assembly Aet v5.0 for *Ae. tauschii* acc. AL8/78. Mis-assemblies in version Aet v4.0 were corrected with optical maps and gaps were closed using contigs assembled from whole-genome-shotgun (WGS) PacBio reads. Transposable elements (TEs) and protein-coding genes were reannotated. The sRNA-producing regions were identified and annotated and their expression was characterized. Selected classes of protein-coding genes and gene families, including abiotic stress genes, were manually annotated. Disease-resistance genes were annotated by a dedicated pipeline.

## Methods

### Optical maps

Seeds of *Ae. tauschii* accessions CIae 1 (lineage 1, Balochistan. Pakistan) and AS75 (lineage 1, Shaanxi province, China) were germinated and grown in the dark. Young leaves were collected for DNA isolation. Ultra-high-molecular-weight (uHMW) DNA was isolated by Amplicon Express (Pullman, WA, USA). DNA was nicked with endonuclease Nt.*Bsp*QI (New England BioLabs, Ipswich, MA, USA), labeled, repaired, and counterstained according to the instructions for the IrysPrep Reagent Kit (Bionano Genomics, San Diego, CA, USA). Consensus optical maps were *de novo* assembled with the Assembler tool (Bionano Genomics, San Diego, CA, USA) using the following significance cutoffs: *P *<* *1 × 10^−9^ to generate draft consensus contigs, *P *<* *1 × 10^−10^ for draft consensus contig extension, and *P *<* *1 × 10^−15^ for final merging of the draft consensus contigs. The initial optical maps were then checked for potential chimeric contigs and accordingly revised.

### Assembly revision with the optical maps

Optical maps were individually aligned to the Aet v4.0 genome assembly, including unanchored sequences to detect mis-assemblies. The alignments were performed with the RefAligner tool (Bionano Genomics, San Diego, CA, USA) with an initial alignment cutoff *P *<* *1 × 10^−10^. Errors in Aet v4.0 indicated by the optical maps were corrected, which generated assembly Aet v4.1.

### Gap closing

Contigs assembled for us from polished whole-genome PacBio SMRT reads of acc. AL8/78 (Zimin *et al.* 2017) by Pacific Biosciences Inc. (Foster City, CA, USA) were aligned to the *Ae. tauschii* acc. AL8/78 optical map (Luo *et al.* 2017), using the RefAligner tool. Chimeric PacBio contigs were corrected. The corrected contigs were then aligned to the pseudomolecules of the Aet v4.1 assembly using the RefAligner tool and their coordinates were recorded. Gaps in Aet v4.1 were closed by replacing Ns with the corresponding PacBio-contig sequence using custom scripts. Closing gaps in assembly Aet v4.1 generated assembly Aet v5.0.

### Transposable element annotation

Transposable elements were annotated following a hybrid strategy consisting of *de novo* TE prediction and homology-based prediction, as described earlier (Luo *et al.* 2017). *De novo* TE prediction employed RepeatModeler (http://www.repeatmasker.org/RepeatModeler, last accessed in 2017), LTR-Finder (Xu and Wang 2007), LTRharvest (Ellinghaus *et al.* 2008), SINE-Finder (Wenke *et al.* 2011), MITE Hunter (Han and Wessler 2010), and Helitron Scanner (Xiong *et al.* 2014). All outputs were manually inspected to eliminate artifacts and combined into a nonredundant custom repeat-sequence library. The Aet v5.0 genome sequence was scanned with this library. The homology-based prediction was performed using RepeatMasker and RepeatProteinMask based on the repeat library from the Repbase database. The TEs annotated by the *de novo* and homology-based methods were merged and redundancies were removed to generate a final TE annotation. Tandem repeats finder (TRF) (Benson 1999) was used to detect tandem repeats in Aet v5.0.

The numbers of complete long terminal repeat-retrotransposons (LTR-RTs) annotated in Aet v4.0 and Aet v5.0 were compared using LTR_finder (Xu and Wang 2007) with default parameters. The output was analyzed with the LTR_retriver pipeline (Ou and Jiang 2018) with default parameters.

### Gene annotation

Gene annotation was performed using *de novo* annotation and homology-based approaches. The latter utilized public RNA-seq assemblies (Hisat2, version 2.0.4, parameter –dta; Stringtie, version 1.2.3, parameters -m 150 -t -f 0.3) (Kim *et al.* 2015; Pertea *et al.* 2015) and inferred putative open-reading frames (ORFs) (Transdecoder, version 3.0.0) (Haas *et al.* 2013). Triticeae protein sequences from available public datasets (UniProt, 05/10/2016) were aligned against the genome sequence using GenomeThreader (version 1.7.1; arguments -startcodon -finalstopcodon -species rice -gcmincoverage 70 -prseedlength 7 -prhdist 4) (Gremme *et al.* 2005). The resulting structure predictions from RNA-seq and protein homology were then merged using Cuffcompare (version 2.2.1) (Ghosh and Chan 2016) to generate a redundant gene set.

*Ab initio* annotation using AUGUSTUS (version 3.3.2) (Stanke *et al.* 2006) was carried out to further improve structural gene annotation. Over-prediction was minimized by generating hint files using the above RNA-seq, protein evidence, and TE predictions. The wheat model was used for prediction.

Structural gene annotations were joined by feeding them into EVidenceModeller (Zhang *et al.* 2008) and weights were adjusted according to the input source. Finally, redundant protein sequences were removed to form a single nonredundant candidate dataset. In order to differentiate among complete and valid genes, noncoding transcripts, pseudogenes, and TEs, blastp (ncbi-blast-2.3.0+, parameters -max_target_seqs 1 -evalue 1e-05) (Altschul *et al.* 1990) was used to compare potential protein sequences with a trusted set of reference proteins (Uniprot Magnoliophyta, reviewed/Swissprot, downloaded on 3 Aug 2016) and PTREP (Release 19), a database of hypothetical proteins that contains deduced amino acid sequences from which, in many cases, frameshifts have been removed, which is useful for the identification of divergent TEs having no significant similarity at the DNA level. Best hits were selected for each predicted protein to each of the three databases. Only hits with an *E*-value below 10e-10 were considered.

Only hits with subject coverage (for protein references) or query coverage (transposon database) above 95% were considered significant. Protein sequences were further classified as HC and LC. An HC protein sequence was a complete sequence that had a subject and query coverage above the threshold in the UniMag database or no blast hit in UniMag, but had a hit in UniPoa and not PTREP. An LC protein sequence was an incomplete sequence that had a hit in the UniMag or UniPoa database, but not in TREP. Alternatively, it could have had no hits in UniMag, UniPoa, or PTREP, but the protein sequence was complete. The tag REP was assigned to protein sequences not in UniMag and complete, but with hits in PTREP.

### Disease resistance gene analog analysis

The protein sequences annotated in Aet v4.0 (Luo *et al.* 2017) were used as inputs into the RGAugury pipeline (Li *et al.* 2016) to identify resistance gene analogs (RGAs). Default database settings (gene3d and Pfam) were applied as query parameters. The distribution of RGAs on the Aet v5.0 pseudomolecules was plotted with the chromosome viewing tool CViT in the R package (Cannon and Cannon 2011). Domain search was performed with the latest interProscan (v85) with the database version 5.51 (Zdobnov and Apweiler 2001).

### Manual annotation

After TE annotation and automated gene annotation, manual annotation of selected genes and gene families followed. To identify gene families, members from a gene family in monocots were used as queries in blastp searches of Aet v5.0 pseudomolecules. The hits were retained along with their coordinates on the Aet v5.0 pseudomolecules. The flanking regions of 5000 bp around each hit were extracted. The FASTA files of these nucleotide sequences were aligned against all corresponding plant gene family members and exons were identified with BLASTX. The individual protein sequences were manually assembled from the blast output. The completed protein sequences were batch blastp searched against plant gene families again to get percent ID values and alignments over the full length of each sequence. Sequences >98% identical were treated as orthologs. Other sequences were named as orthologs if the percent identity of their next best blast hit was significantly less than that of the best blast hit. Allowances were made for indels that would lower the percent identity, if the aligned parts were highly similar. Sequences that did not meet this requirement were assigned nonorthologous names. Some sequences were identical (or nearly identical) duplicate copies either adjacent to each other or in more distant locations, even on other chromosomes. These sequences were named with an *a* or *b* extension of the name.

To identify the cold- and frost-tolerance-related genes and gene families implicated in abiotic stress responses or G protein signaling in *Ae. tauschii*, gene sets of CBF, dehydrin, and ICE genes from other cereals were used. These genes were used in BLAST searches against the Aet v5.0 assembly and the resulting hits were individually characterized. The exon-intron boundaries and the UTR regions were determined by using ESTs (NCBI, JCVI) TSAs (NCBI) and transcriptomic RNA-seq data (European Nucleotide Archive). The NCBI BLAST server and Pfam (HMM) domain search were used for functional annotations.

### Small RNA analysis

RNA was extracted from roots, spikes, and leaves of large plants and from whole seedlings of AL8/78 using TriReagent (Millipore-Sigma, St. Louis, USA) following manufacturer’s protocols. The sRNAs were annotated using newly generated sRNA libraries (GSE169198). Libraries were generated using 20 ng of RNA after size selection on polyacrylamide gels, and using the NEBNext Small RNA Library Prep Set for Illumina (New England Biolabs, Ipswich, USA) (Mathioni *et al.* 2017). Libraries of sRNAs were processed as previously described (Mathioni *et al.* 2017). Briefly, library quality was assessed using FastQC (http://www.bioinformatics.babraham.ac.uk/projects/fastqc/), adapters were trimmed using Trimmomatic v0.32 (Bolger *et al.* 2014) with a minimum and maximum length of 18 and 34 nt, respectively, and mapped with Bowtie2 (Langmead and Salzberg 2012). The miRNA predictions were done using both ShortStack (Johnson *et al.* 2016) and miR-PREFeR (Lei and Sun 2014) and filtering criteria recommended in Axtell and Meyers (Axtell and Meyers 2018). PhasiRNAs were annotated using ShortStack (Johnson *et al.* 2016) and a filtering phasing score of 25. Hc-siRNA prediction was done by Bowtie mapping to the newly annotated TEs, using only small RNAs of 21 to 24 nt long. The tRF prediction was done by identifying reads mapped by Bowtie to the newly annotated tRNAs. Plots were produced using R gplot and ggplot2 (Wickham 2009).

## Results and discussion

### Revisions of super-scaffolds with optical maps

**Figure 1:**
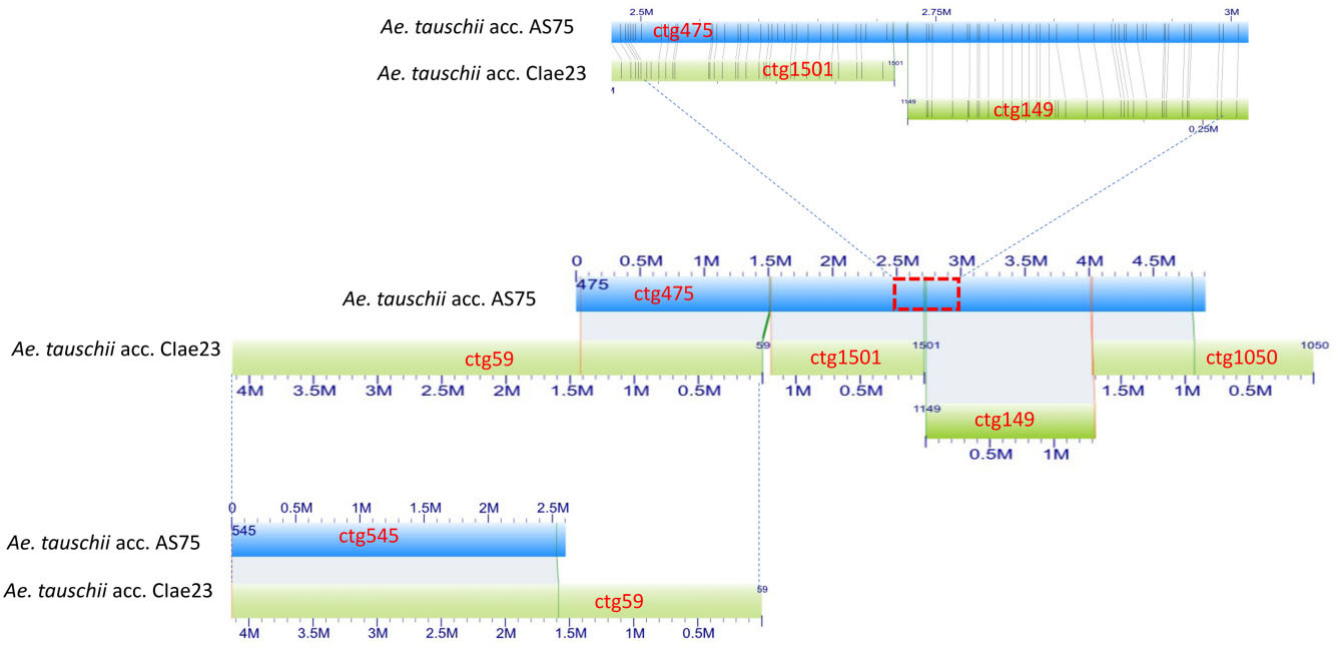
Differences in gap distribution on optical maps for different *Ae. tauschii* accessions. (Middle) Four separate optical contigs (ctg59, ctg1501, ctg149, and ctg1050) of *Ae. tauschii* accession CIae 23 (lineage 2) (green rectangles) aligned on a single optical contig (ctg475) of *Ae. tauschii* accession AS75 (lineage 1) (blue rectangles). (Top) A detail of the alignment of optical contigs ctg1501 and ctg149 on optical contig ctg475. The vertical lines connect corresponding restriction sites in aligned contigs. (Bottom) An overlap between AS75 optical contig ctg454 and CIae 23 optical contig ctg59 closes a gap between AS75 optical contigs ctg475 and ctg454 and extends the contiguity of optical map alignments.

Genome-wide optical maps were constructed for acc. CIae 1 and acc. AS75. The total lengths of the maps were 4.29 and 4.69 Gb with a contig N50 of 1.72 and 1.12 Mb, respectively (Table 1). As anticipated, some gaps between contigs of the two optical maps differed from those of the lineage 2 optical maps used for assembly Aet v4.0 (Figure 1).

**Table 1:** Metrics for the genome-wide optical maps of *Aegilops tauschii* acc. CIae 1 and AS75

Metric	CIae 1	AS75
Contigs (no.)	3603	5507
Map total length (Gb)	4.29	4.69
Contig N50 (Mb)	1.72	1.12
Max contig length (Mb)	14.66	12.62

The AS75 and CIae 1 optical contigs were separately aligned to the Aet v4.0 genome assembly including unanchored scaffolds to search for mis-assemblies. Two super-scaffolds with a total length of 3.98 Mb on pseudomolecules Chr2 and Chr7 were found to be incorrectly placed. Their placement and/or orientation were revised. This revision produced assembly Aet v4.1.

### Closing gaps with PacBio contigs

**Figure 2:**
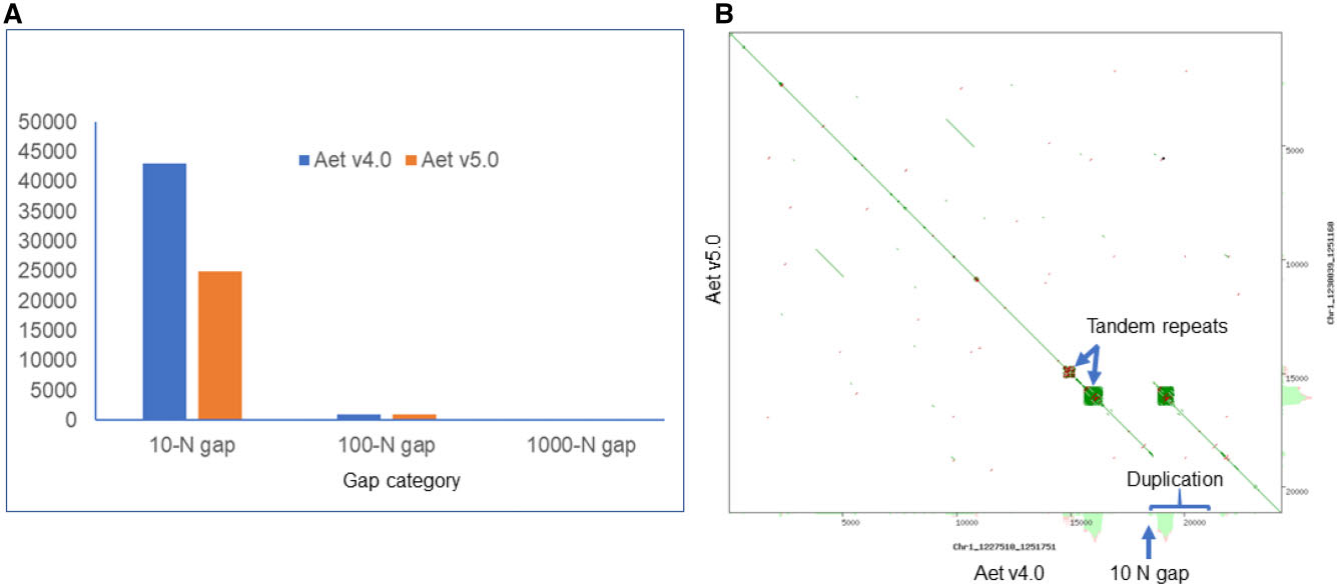
Gaps in the Aet v4.0 and v5.0 assemblies. (A) Number of gaps filled with 10, 100, and 1000 Ns in the two assemblies. (B) A dot plot showing details of a 10-N gap and duplication created by erroneous scaffold assembly in two regions harboring tandem repeats in pseudomolecule Chr1 of Aet v4.0, region 1,227,510–1,251,751 bp.

Corrected PacBio contigs were used to close gaps by replacing Ns with actual PacBio sequences. Aet v4.1 contained the gaps present in the original assembly Aet 4.0. The gaps were both of predicted and unknown lengths. The former gaps contained numbers of Ns corresponding to the lengths of gaps in bp. The latter gaps contained 10, 100, or 1000 Ns as placeholders; 10 Ns were inserted by NRGene to link contigs, 100 Ns were inserted by the Bionano pipeline during hybrid scaffolding, and 1000 Ns were used to link adjacent scaffolds and super-scaffolds during the construction of Aet v4.0 pseudomolecules (Luo *et al.* 2017). Of these three classes, gaps filled with 10 Ns were most abundant (Figure 2A). Gap filling reduced the number of 10-N gaps from 43,066 in Aet v4.0 to 24,973 in the updated assembly, Aet v5.0 (Figure 2A). Since gap closing only replaced Ns with actual nucleotide sequences, the statistics of the Aet v5.0 assembly, such as the super-scaffold N50 value, number of super-scaffolds, and their minimum and maximum lengths, were barely changed compared to Aet v4.0.

Some of the 10-N gaps were not real gaps, but assembly errors. An example of such an error is shown in Figure 2B. The assembly of a region terminating in a block of tandem repeats generated an erroneous segmental duplication, which duplicated the region including the block of tandem repeats in Aet v4.0. Our removal of the duplication and the 10-N gap shortened the scaffold length. Overall, the total length of the Aet v5.0 pseudomolecules was reduced by 3.77 Mb compared to those of Aet v4.0 assembly (Table 2). At the same time, the effective length (the length of an assembly without Ns) of the Aet v5.0 pseudomolecules increased by 9.69 Mb (Table 2).

**Table 2:** Comparison of characteristics of the Aet v4.0 and v5.0 pseudomolecules

Pseudo-molecule		Total length (bp)	Length of gaps (bp)	Effective length (bp)	Gap number
Chr1	Aet v4.0	502,330,251	10,377,979	491,952,272	11,744
	Aet v5.0	501,967,303	8,406,331	493,560,972	6,647
Chr2	Aet v4.0	651,661,114	10,780,418	640,880,696	14,522
	Aet v5.0	650,458,083	8,558,830	641,899,253	8,139
Chr3	Aet v4.0	627,182,665	11,118,990	616,063,675	14,404
	Aet v5.0	627,456,150	9,202,775	618,253,375	9,437
Chr4	Aet v4.0	526,018,785	9,454,858	516,563,927	10,892
	Aet v5.0	525,206,139	8,086,205	517,119,934	6,175
Chr5	Aet v4.0	577,375,663	14,628,972	562,746,691	13,506
	Aet v5.0	576,238,907	12,629,354	563,609,553	7,615
Chr6	Aet v4.0	496,019,527	9,112,991	486,906,536	11,357
	Aet v5.0	495,363,004	7,581,178	487,781,826	6,199
Chr7	Aet v4.0	644,716,137	17,324,389	627,391,748	15,384
	Aet v5.0	644,841,383	14,867,162	629,974,221	8,698
Total	Aet v4.0	4,025,304,142	82,798,597	3,942,505,545	91,809
	Aet v5.0	4,021,530,969	69,331,835	3,952,199,134	52,910
Difference		−3,773,173	−13,466,762	9,693,589	−38,899

### Transposable element annotation

Repeated sequences represented 86.88% of the genome sequence in Aet v5.0 (Table 3), exceeding the 84.4% estimated earlier (Luo *et al.* 2017). The increase was due to the more contiguous nature of the Aet v5.0 sequence and reduction in the total length of the assembly.

**Table 3:** Numbers, total length, and percentages of the Aet v5.0 genome sequence represented by classes of transposable elements

Class	Elements (no)	Sequence length (bp)	Genome length (%)
Class I			
LTR/Gypsy	853,285	1,333,833,165	33.17
LTR/Copia	383,555	644,052,368	16.02
LTR/Unknown	456,559	611,381,110	15.20
Non-LTR/LINE	192,648	80,369,034	2.00
Non-LTR/SINE	19,503	3,344,990	0.08
Class II			
CACTA	201,749	203,285,995	5.05
Mutator	29,706	10,397,597	0.26
PIF-Harbinger	77,072	22,128,071	0.55
Tc1-Mariner	166,095	48,513,995	1.2
hAT	17,127	3,870,055	0.09
Helitron	219,796	113,044,338	2.81
MITE/Stowaway	1,139	174,827	0.00
MITE/Tourist	453	80,546	0.00
DNA/unknown	1,245	296,555	0.01
Unspecified	393,632	410,074,883	10.20
Total interspersed	3,013,564	3,484,847,529	86.65
Low complexity	20,328	1,104,950	0.03
Simple repeat	173,933	7,922,189	0.20
Total	3,207,825	3,493,874,668	86.88

Long terminal repeat-retrotransposons were the most abundant class of TEs, accounting for 64.39% of the genome sequence (Table 3). Of them, *Gypsy* LTR-RTs were most abundant, representing 33.17% of the genome sequence. CACTA elements were the most abundant DNA TEs and accounted for 5.05% of the genome sequence.

One of the results of closing gaps was annotation of a greater number of intact TEs in the genome sequence, particularly LTR-RTs. Some 50,733 intact LTR-RTs were identified in Aet v4.0, but 57,076 were identified in Aet v5.0 (Table 4). This increase was observed in all LTR-RT classes (Table 4).

**Table 4:** Comparison of complete LTR-retrotransposons in the Aet v4.0 and v5.0 pseudomolecules

Pseudomolecule	*Copia*		*Gypsy*		Unknown	
	Aet v4.0	Aet v5.0	Aet v4.0	Aet v5.0	Aet v4.0	Aet v5.0
Chr1	1,674	1,941	3,139	3,530	1,637	1,795
Chr2	2,030	2,434	3,922	4,427	2,184	2,375
Chr3	1,942	2,254	3,777	4,091	2,067	2,197
Chr4	1,625	1,905	3,461	3,879	1,736	1,884
Chr5	1,975	2,392	3,485	3,888	1,890	2,099
Chr6	1,603	1,931	3,019	3,429	1,663	1,799
Chr7	2,081	2,439	3,689	4,080	2,134	2,307
Total	12,930	15,296	24,492	27,324	13,311	14,456

### Gene annotation

#### Automated annotation

A total of 39,635 HC protein-coding genes was annotated in assembly Aet v4.0. This number was similar to the 39,647 genes annotated by Zhao *et al.* (2017) in their *Ae. tauschii* acc. AL8/78 sequence. Using a combination of *de novo* and homology-based methods, our pipeline annotated 32,885 and 35,903 HC and LC protein-coding genes, respectively, in Aet v5.0 (Table 5). The number of HC genes annotated in Aet v5.0 was lower by 6750 genes compared to the number of HC genes annotated in Aet 4.0 (Table 5). The number of HC genes was slightly lower than the number of genes recently annotated in other genome assemblies in Triticeae. For instance, 34,265 HC genes were annotated in the D subgenome of Chinese Spring wheat IWGSC RefSeq 2.1 (Zhu *et al.* 2021) and 35,827 HC genes were annotated in barley assembly Morex v3 (Mascher *et al.* 2021).

**Table 5:** High-confidence (HC) and low-confidence (LC) genes generated by automated annotation in the Aet v4.0 and Aet v5.0 assemblies

Metric	Aet v4.0		Aet v5.0	
	HC	LC	HC	LC
Total genes (no.)	39,635	43,495	32,885	35,903
Single-exon genes (no.)	15,389	36,567	9,837	25,186
Multi-exon genes (no.)	24,246	6,928	23,048	10,717
Mean CDS length (bp)	1,133	319	1,250	593
Median CDS length (bp)	942	258	1,071	435
Mean exons per transcript (no.)	3.9	1.2	4.45	1.68
Median exons per transcript (no.)	2	1	3	1
Missing BUSCO genes	93		50	

If the lower number of HC genes annotated in Aet v5.0 was an artifact caused by too-stringent conditions implemented in the annotation pipeline, it is expected that the number of BUSCO genes present in the Aet v5.0 annotation would also decline compared to the Aet v4.0 annotation. Based on 1440 BUSCO v3.1 single-copy genes (Simão *et al.* 2015), annotation completeness actually improved in Aet v5.0 compared to Aet v4.0 (Table 5). A total of 1374 genes were detected as a single copy (1350) or duplicated (24) genes in Aet v5.0, while 1289 BUSCO genes (1268 single copy and 21 duplicated) were found in Aet v4.0. Gene fragments of 16 genes were found in Aet v5.0 compared to 58 in Aet v4.0. Fifty BUSCO genes were missing in Aet v5.0, but 93 were missing in Aet v4.0. The 96.5% BUSCO gene coverage in Aet v5.0 was comparable to 97% presence in the *Ae. tauschii* AL8/78 assembly reported by Zhao *et al.* (2017). A slightly greater percentage of BUSCO genes was present in the most recent barley (Morex v3) annotation (98.6%) (Mascher *et al.* 2021), but this comparison should be treated with caution since fewer BUSCO genes (425) were used in the barley annotation assessment compared to the 1440 used by us.

We also assessed whether genes present in Aet v4.0 but absent in Aet v5.0 were present among HC genes in the most recent wheat genome reference sequence assembly, IWGSC RefSeq 2.1. This comparison was made for the annotation of *Ae. tauschii* pseudomolecule Chr1. There were 4998 HC and 5405 LC genes annotated on pseudomolecule Chr1 in Aet v4.0. Of them, 917 HC and 4673 LC genes were absent from Aet v5.0. Of the absent genes, only 163 HC (about 3%) and 43 LC (<1%) genes were present in annotation of IWGSC RefSeq 2.1. Based on these data, about 3% protein-coding genes may have been missed in the Aet v5.0 annotation. Since 90% sequence identity was used in these searches, the actual number of genes missed in Aet v5.0 may be even smaller, since some paralogues in IWGSC RefSeq 2.1 could have been mistaken for orthologues. This characterization suggests that gene annotation may have been insufficiently stringent in Aet 4.0. The abundance of 10-N gaps in Aet v4.0, some falling inside genes, could have resulted in annotating pseudogenes or gene fragments as *bona fide* genes. Both BUSCO gene analysis and the characterization of missing genes annotated in pseudomolecule Chr1 suggested that the Aet v5.0 assembly annotation is of higher quality than the Aet v4.0 assembly annotation.

#### Manual annotation

A total of 2822 manual gene annotations was made (Supplementary Table S1). After eliminating duplications, the database contained manual annotation of 2245 genes, which included functionally characterized genes affecting wheat phenology, such as *VRN1* (Chen and Dubcovsky 2012), *VRN2* [*ZZCT1* and *ZZCT2*, (Yan *et al.* 2004)], *VRN3 = FT1* (Yan *et al.* 2006), *FT2* (Shaw *et al.* 2019), *PPD1, CO1*, and *CO2* (Shaw *et al.* 2020), *PHYC* and *PHYB* (Chen *et al.* 2014), and *ELF3* (Alvarez *et al.* 2016) (Supplementary Table S1 at Budak and Dubcovsky tabs) and wheat end-use quality, such as prolamin genes (Huo *et al.* 2018a, 2018b). There were 2, 3, 4, 5, and 2 genes for δ-gliadins, γ-gliadins, ω-gliadins, low-molecular-weight glutenins, and high-molecular-weight glutenins, respectively, on pseudomolecule Chr1 and 13 genes for α-gliadins on pseudomolecule Chr6 (Supplementary Table S1 at Gu tab). Eleven of these 32 *Ae. tauschii* prolamin genes were pseudogenes or gene fragments (Supplementary Table S1 at Gu tab).

The manual gene annotations also included abiotic stress and G protein signaling genes, such as heterotrimeric G proteins, caleosins, phospholipases (Khalil *et al.* 2011, 2014) (Supplementary Table S1 at Budak, Gulick, and Galiba tabs), and several important families of transcription factors (TFs) (Supplementary Table S1 at Budak tab). Abiotic stress responsive genes included Early Salt stress Induced (ESI) genes (Brunetti *et al.* 2018) and dehydrin genes. Dehydrins include subtypes induced by drought, salinity, and/or cold stresses with tissue and genotype-specific expression profiles (Wang *et al.* 2014b). The large DREB (dehydration-responsive element binding) family includes subgroups responsive to different stress factors, usually with tissue-specific expression profiles (Supplementary Table S1 at Budak and Galiba tabs). DREB1 proteins respond to cold stress while DREB2 proteins are upregulated by drought and salt stresses. Other TF families manually annotated in Aet v5.0 were general regulators of stress responses. One such family, the NAC family, is involved in metabolic processes including abiotic and biotic stress responses (Supplementary Table S1 at Budak tab).

A total of 552 cytochrome P450 (CYP) genes were manually annotated in Aet v5.0 and compared with those annotated in wheat (Supplementary Table S1 at Nelson tabs). CYP genes are members of one of the largest gene superfamilies in plant genomes (Nelson and Werck-Reichhart 2011). They play roles in numerous plant developmental processes and are involved in multiple metabolic pathways including defense against biotic and abiotic stresses.

Finally, 1921 RGAs were identified in Aet v5.0 with a dedicated pipeline (Supplementary Table S1 at You tab). This number was similar to the 2243 identified in the wheat (cv Chinese Spring) D subgenome (Table 6). RGAs were categorized as nucleotide-binding site domain (NBS) genes, receptor-like protein kinase (RLK) genes, receptor-like protein (RLP) genes, and transmembrane coiled-coil protein (TM-CC) genes (Table 6). The absence of Toll/interleukin-1 receptor-like domain (TNL) genes both in *Ae. tauschii* and the wheat D subgenome is consistent with the hypothesis that the TNL type of NBS coding genes did not evolve in monocots or were lost (Meyers *et al.* 1999; Pan *et al.* 2000; Akita and Valkonen 2002; Bai *et al.* 2002; Cannon *et al.* 2002).

**Table 6:** Comparison of numbers of resistance gene analogues (RGAs) identified in the *Ae. tauschii* genome and the D subgenome of wheat (*T. aestivum* cv Chinese Spring)

	RGAs^*a*^								
	NBS	CNL	TNL	CN	TN	NL	RLP	RLK	TM-CC
*Ae. tauschii*	119	215	0	85	3	221	145	991	142
*T. aestivum^b^*	145	235	0	105	4	280	206	1094	174

a NBS, nucleotide-binding site domain; LRR, leucine-rich repeat; TNL, Toll/interleukin-1 receptor-like domain; CNL, CC–NBS–LRR; TNL, TIR-NBS-LRRs; TN, TIR–NBS; RLK, receptor-like protein kinase; RLP, receptor-like protein; TM-CC, transmembrane coiled-coil protein.

b Computed from Mayer *et al.* (2014).

**Figure 3:**
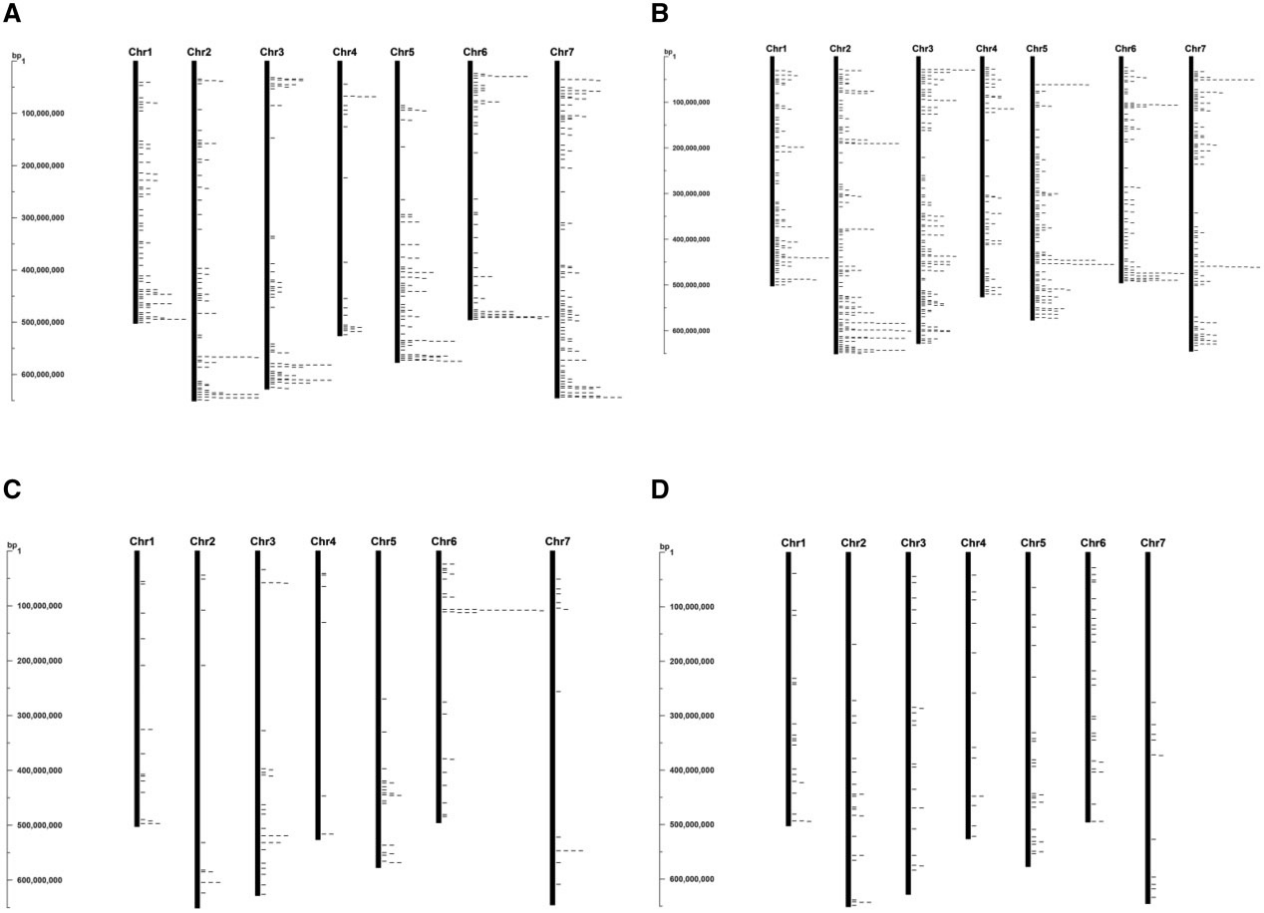
Distribution of resistance gene analogs (RGAs) along the *Ae. tauschii* pseudomolecules. Genes encoding (A) nucleotide-binding site domains (NBS), (B) receptor-like protein kinases (RLK), (C) receptor-like proteins (RLP), and (D) transmembrane coiled-coil proteins (TM-CC) in the Aet v5.0 genome sequence are indicated by ticks to the right of the chromosome bars. A registry in bp is shown to the left of each figure.

Most of the NBS, RLP, and RLK coding genes were clustered at the ends of the *Ae. tauschii* chromosomes (Figure 3, A, B, and C). In contrast, the TM-CC genes were more evenly dispersed along the chromosomes (Figures 3D). These two types of RGAs differed in that the NBS, RLP, and RLK genes were often in multi-copy loci, whereas most of the TM-CC genes were in single-copy loci (Figure 3). The difference in the distribution of NBS, RLP and RLK genes on one hand and TM-CC genes on the other hand was likely due to the location preference of multi-copy loci for distal, high-recombination chromosomal regions in Triticeae (Akhunov *et al.* 2003; Luo *et al.* 2017).

Two tracks were added to the Aet v5.0 JBrowse to integrate the manual gene annotations (http://aegilops.wheat.ucdavis.edu/jbrowse/index.html?data=Aetv5). One track includes the RGA genes annotated by the dedicated pipeline and the other includes the manually annotated genes.

### Small RNA annotation

**Figure 4:**
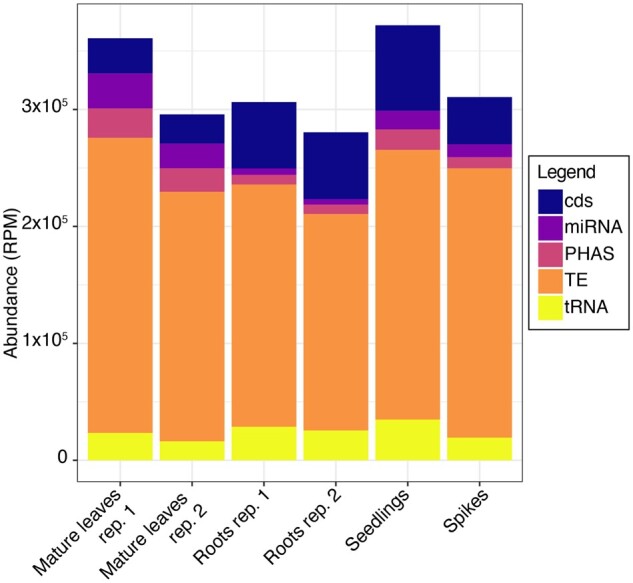
Abundance of sRNAs in *Ae. tauschii* tissues. Blue, purple, orange, ochre, and yellow represent sRNAs derived from CDS, miRNAs, phasiRNAs, TE-derived sRNAs, and tRNA-derived sRNAs, respectively.

Small RNAs were isolated and characterized from two samples of mature leaves, two samples of roots, and one sample each from whole seedlings and spikes of *Ae. tauschii* acc. AL8/78. In each sample, TE-derived sRNAs (hc-siRNAs) were most abundant (Figure 4). Next most abundant were miRNAs and tRFs, which each represented approximately 2.5 to 5.0% of the sRNA population (Figure 4). Sizes of sRNAs peaked at 24 nt with a secondary peak at 21 nt in all six samples (Supplementary Figure S1). When sRNAs of the same lengths but different sequence (distinct sRNAs) were considered, only the 24-nt peak was observed (Supplementary Figure S1). This difference was due to the low diversity of 21-mers, since they were mostly comprised of a small number of abundant miRNAs, and the high diversity of 24-mers, being mainly composed of hc-siRNAs.

#### miRNAs

These sRNAs are typically 21 or 22 nt long and are derived from hair-pin single-strand miRNA precursors (pre-miRNAs). Using two different prediction tools and stringent filtering criteria, 61 pre-miRNAs were identified (Supplementary Table S2). Pre-miRNAs were located on all seven pseudomolecules and had an average length of 150 nt with a range from 76 to 296 nt (Supplementary Table S2). The pre-miRNAs produced 124 mature miRNAs belonging to 38 miRNA families, of which 27 were known families and 11 were new candidates. In all six samples, the peak length of mature miRNAs was 21 nt (Supplementary Figure S2). A total of 177 target genes were detected for the 124 mature miRNAs (Supplementary Table S3).

Compared to the 124 we found in our experiments, nearly twice as many miRNAs (238) were predicted by homology search of the *Ae. tauschii* AL8/78 genome sequence (Zhao *et al.* 2017). Homology searches of *Ae. tauschii* RNA-seq databases predicted miRNAs belonging to 55 families (Alptekin and Budak 2017), compared to 38 families that we experimentally uncovered in our study. These differences are likely an outcome of the limited numbers of tissues included in our *de novo* search approach.

Most of the miRNAs identified in our study were intergenic, six were intronic, and four were in untranslated regions of genes (Supplementary Table S2). The miRNAs tend to be clustered in the genome (Boualem *et al.* 2008; Barik *et al.* 2014) and some of these clusters may be transcribed as a single transcriptional unit, named polycistronic miRNAs. Here, pre-miRNAs with a genomic location separated by <3000 nt were considered as potentially polycistronic miRNAs. Based on this criterion, four potential polycistronic miRNAs (Supplementary Table S2) were defined, two of them with folding structures that had miRNA characteristics (Supplementary Figure S2).

**Figure 5:**
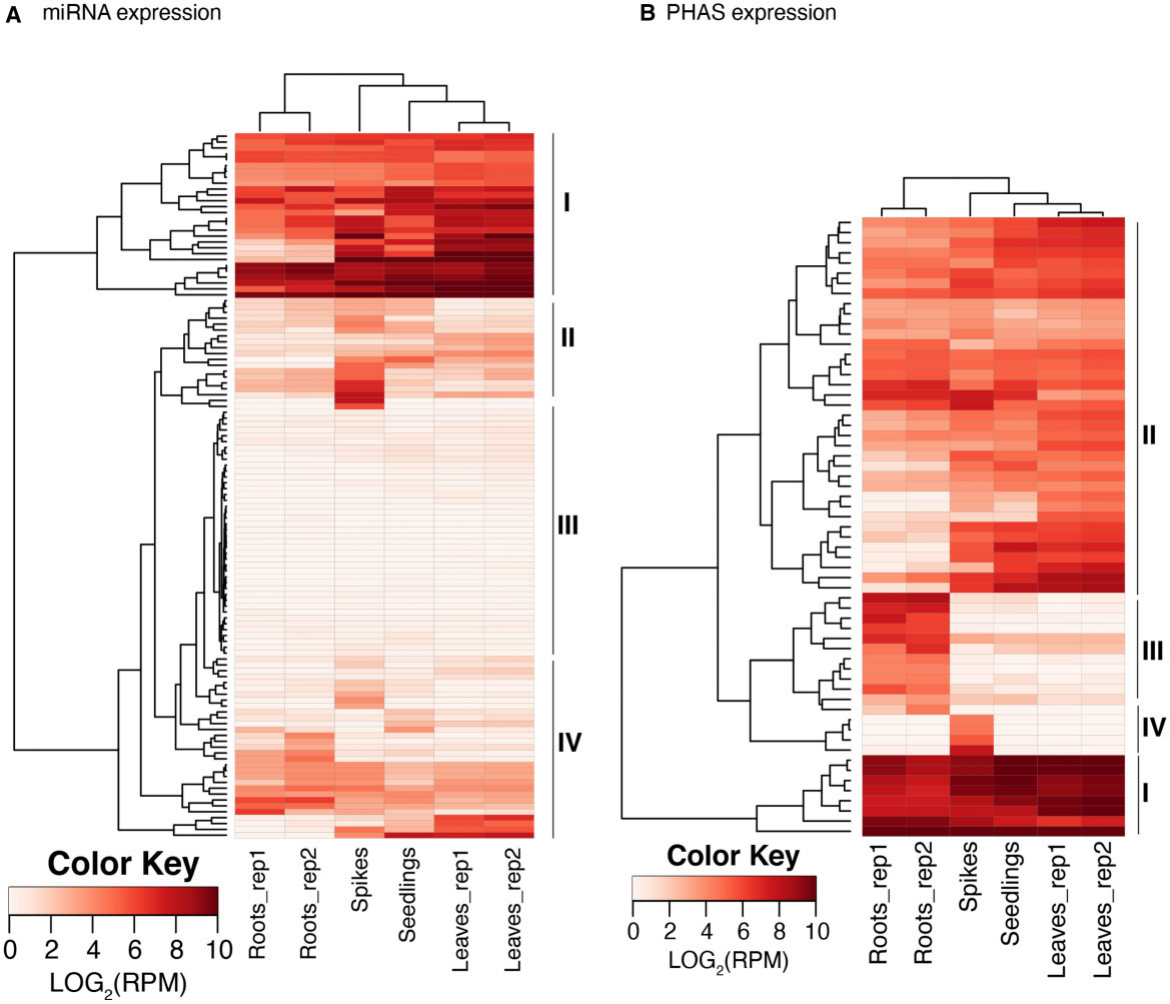
miRNA and phasiRNA expression. Heatmaps of miRNA (A) and phasiRNA (B) expression in different *Ae. tauschii* tissues. The rows represent mature miRNAs and *PHAS* loci, respectively, and the columns represent samples from mature leaves (2 replicates), roots (2 replicates), seedlings, and spikes. The miRNAs and *PHAS* loci were grouped into four different clades (I–IV) based on the expression level.

Based on the accumulation level, mature miRNAs were grouped into four different clades (Figure 5A). Clades I, II, and III were high, moderate, and low abundance, respectively, within each of the six RNA samples. Mature miRNAs of clade II accumulated at higher levels in spikes. Clade IV miRNAs had a highly variable accumulation levels across the sampled tissues.

#### phasiRNAs

These sRNAs are 21 or 24 nt long, originate from coding or noncoding regions, and are typically triggered by 22-nt long miRNAs. A total of 60 *PHAS* loci, 57 corresponding to coding genes and three corresponding to noncoding genes, were identified. All *PHAS* loci identified in this study produce mainly 21-nt phasiRNAs (Supplementary Table S4). As previously described (Jeong *et al.* 2013), most of the coding *PHAS* loci encoded disease resistance proteins (37 out of 59, 62%). Other coding *PHAS* loci included genes encoding protein kinases, auxin response factors, and uncharacterized proteins (Supplementary Table S4). Of the noncoding *PHAS* loci, two loci corresponded to *TAS3* loci (Supplementary Table S4). This is fewer than the four identified in noncoding loci by previous genomic analysis (Xia *et al.* 2017), perhaps reflecting an absence of sRNAs from two loci in the tissues investigated. This is a surprisingly low number given that other plant genomes contain up to 70 noncoding *PHAS* loci (Xia *et al.* 2017). Using the target prediction tool psRNAtarget (Dai *et al.* 2018b), miR390-5p was identified as the trigger of *TAS3* phasiRNAs (Supplementary Table S5). We identified miR9863-3p as the putative trigger of most of the defense proteins (Supplementary Table S5).

Based on their accumulation levels, phasiRNAs could be grouped into four different clades (Figure 5B). Clades I and II have similar accumulation levels in all samples; clade I had a high accumulation and clade II had a moderate accumulation. Clade III phasiRNAs had a specific high accumulation in roots and clade IV had a specific accumulation in spikes.

#### hc-siRNAs

**Figure 6:**
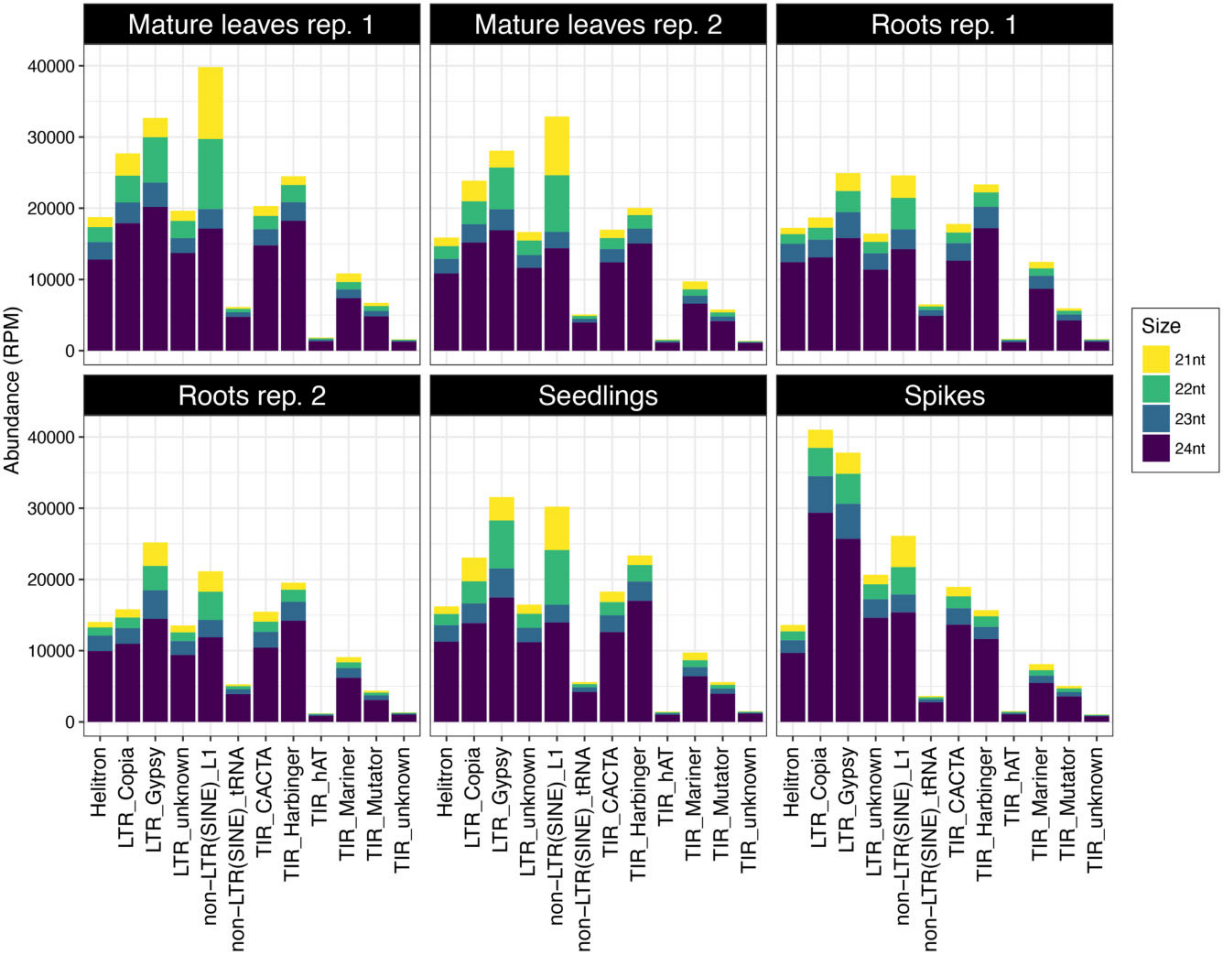
Composition and size distribution of hc-siRNA in *Ae. tauschii.* The hc-siRNAs were categorized by their size and their source in mature leaves (2 replicates), roots (2 replicates), seedlings, and spikes. Yellow, green, cyan, and purple bars represent hc-siRNAs 21, 22, 23, and 24 nt long, respectively.

These are 21 to 24-nt sRNAs that map to repetitive regions of the genome. They typically map to many locations, confounding the identification of their precise origin. The majority of hc-siRNAs were 24 nt long, with substantially lower levels of those 23, 22, and 21 nt in length (Supplementary Figure S1). The majority of hc-siRNAs were derived from nonLTR SINE elements and *Gypsy and Copia* LTR-RTs (Figure 6). Differences among tissues regarding the hc-siRNAs origin were observed (Figure 6). For example, *Copia-*derived hc-siRNAs were the most abundant class in spikes but not in other tissues.

#### tRFs

**Figure 7:**
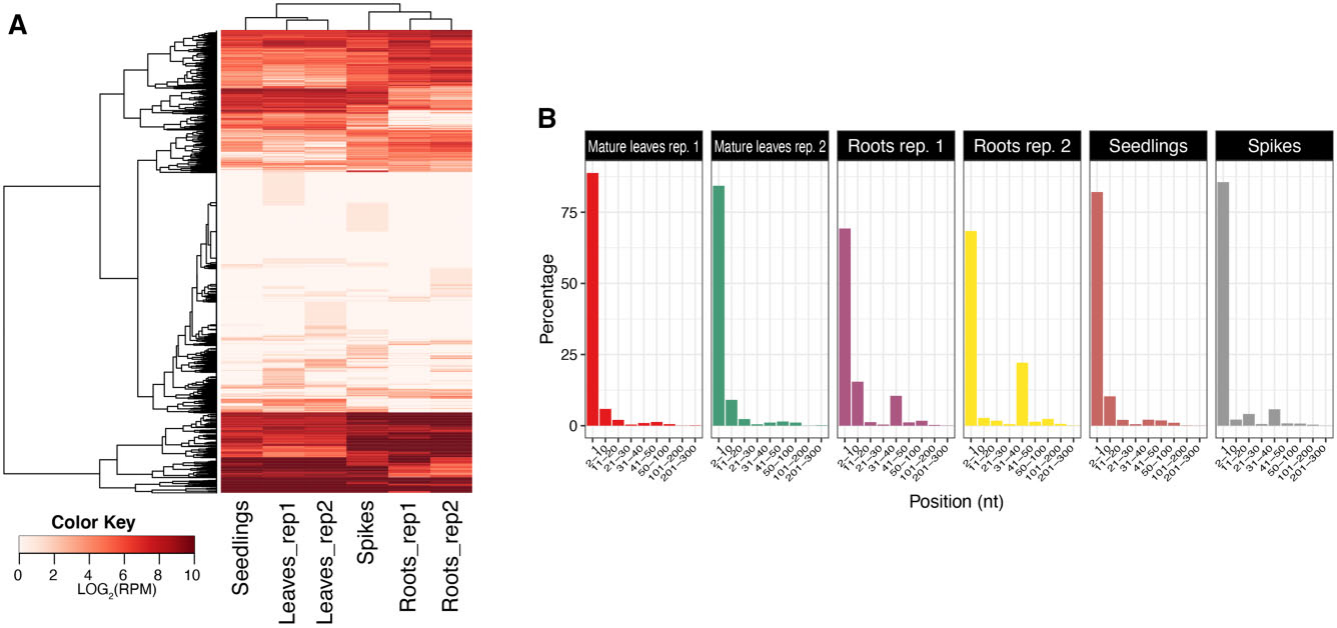
Characterization of tRNA-derived fragments. (A) Heatmap of tRNA-derived fragments in *Ae. tauschii* tissues. The rows of the heat map represent tRNA genes and the columns represent samples. (B) Origins of the tRNA-derived fragments in the six *Ae. tauschii* samples. The tRFs are classified depending on their original position within the tRNA molecule. The *x*-axis represents the positions within each tRNA (divided into nine different bins) and the *y*-axis represents the abundance (in %).

These sRNAs are 19 to 34 nt long and derived from tRNA precursors that may act much like miRNAs, particularly in post-transcriptional regulation of gene expression. They displayed three major lengths: 19, 26, and 32 nt (Supplementary Figure S1). Regardless of their origin or classification, their accumulation was consistent among the RNA samples (Figure 7A). The tRFs may be classified as: 5-tRF, originating from the 5' end, i-tRF, originating from internal tRNA regions, and 3-tRF, originating from the 3' end. To analyze the abundance of these three tRF categories, the 5' position of each tRF for each sample was plotted, and to simplify the visualization, the positions within each tRF were allocated into nine different bins reflecting typical cleavage positions (Keam and Hutvagner 2015). The majority of tRFs, between 68% and 88% depending on the sample, were 5-tRFs originating from the first nucleotide of the tRNA molecule (Figure 7B). The second most abundant category was i-tRFs.

## Conclusions

The deployment of optical maps for *Ae. tauschii* accessions from phylogenetic lineage 1 (Wang *et al.* 2013) facilitated bridging gaps on our previous optical maps. Two erroneous assemblies present in pseudomolecules Chr2 and Chr7 in Aet v4.0, equivalent to 3.98 Mb and representing 0.09% of the total lengths of the pseudomolecules, were identified and rectified in Aet v5.0.

An additional improvement of the Aet v5.0 assembly was achieved by closing 38,899 gaps with PacBio contigs. Replacement of Ns with actual sequences increased the effective sequence length by 9.69 Mb (0.2%), but also produced minor structural changes in the sequence assembly, as illustrated in Figure 2B. The majority of gaps in Aet v4.0 were filled with 10-N designations as placeholders during the WGS sequence assembly. Since most of the 10-N gaps were artifacts, replacements of Ns by PacBio sequences actually reduced the total length of the pseudomolecules by 3.77 Mb, to 4.021 Gb. In addition, 199 Mb are in unassigned scaffolds in the Aet v5.0 assembly. The shorter total length of the sequence and its greater contiguity increased the estimated repetitive portion of the genome from 84.4% to 86.9%.

The number of predicted protein-coding genes decreased from 83,130 in Aet v4.0 to 68,788 in Aet v5.0. The numbers of single exon gene models decreased by 6749 (43.8%) for the HC gene models and by 7592 (29.8%) for the LC gene models. In contrast, the number of multi-exon HC gene models was reduced by only 1198 (4.9%) while the number of multi-exon LC gene models increased by 3789 (35.4%). This conspicuous abundance of single-exon gene models in Aet v4.0 may have been caused by 10-N gaps erroneously joining different exons into a single artifactual exon, resulting in an overestimation of single-exon gene models. Improvements in the annotation pipeline could be another cause.

A total of 2245 genes was annotated either manually or by a dedicated pipeline in Aet v5.0. This annotation focused on genes of known function in wheat, environmental stress tolerance genes, TFs, some large gene superfamiles, such as CYP encoding genes, and RGAs. Manual annotation and RGA tracks were included into Aet v5.0 JBrowse to displays these genes. The tracks will be updated as the understanding of the function of genes in *Ae. tauschii* grows.

New in the Aet 5.0 assembly is the annotation and analysis of sRNAs. The analysis showed that most of the sRNAs were TE-derived hc-siRNAs. A total of 61 pre-miRNAs producing 124 mature miRNAs were *de novo* identified in the Aet v5.0 sequence by sequencing sRNA from roots, leaves, whole seedlings, and spikes. In addition, 60 phasiRNAs were detected; most of them (62%) were derived from disease resistance genes.

Despite these advances in the assembly and annotation of the *Ae. tauschii* acc. AL8/78 genome, more remains to be done. Discrepancies below 10 kb were below the resolution of optical maps and could not be detected. A total of 199 Mb of scaffolds remains unplaced. The sequences of the centromeric regions and regions containing tandem repeats have been inadequately assembled or are absent. Nevertheless, the advances featured in Aet v5.0 make this assembly a more valuable resource for wheat science, biotechnology, and breeding.

## Data availability

Aet v5.0 assembly is available at NCBI under Bioproject PRJNA341983. A JBrowse (http://aegilops.wheat.ucdavis.edu/jbrowse/index.html?data=Aetv5) was constructed to present Aet v5.0 and annotations (including manual annotation). The small RNA raw and processed data have been deposited into the Gene Expression Omnibus under the accession code GSE169198. Supplementary material is available at figshare: https://doi.org/10.25387/g3.16567566.

## Author contributions

J.Dv., M.-C.L., K.M.D., P.E.M., L.W., and T.Z. conceived the project. T.Z., J.C.R., K.R.D., and M-C.L. generated optical maps. T.Z. and M.-C.L. validated the assembly with optical maps. L.W., T.Z., and M.-C.L. performed gap closure with PacBio contigs. T.L., M.S., and K.F.X.M. re-annotated genes and H.Z., K.M.D., L.W., and J.L.B. re-annotated TEs. P.B. and B.C.M. analyzed small RNAs. N.H., Y.Q.G., J.Du., G.G., B.K., D.N., T.U., H.B., and P.J.G. conducted manual annotations. P.L. and F.M.Y. annotated RGAs. L.W., T.Z., P.B., M-C.L., P.E.M., and J.Dv wrote the first draft of the paper. All authors edited and approved the final draft.

## Funding

This material is based upon work supported by the National Science Foundation under Grant No. IOS-1238231.

## Conflicts of interest

The authors declare that there is no conflict of interest.
